# Efficacy and Safety of Direct Oral Anticoagulants for Secondary Prevention of Cancer-Associated Thrombosis: A Systematic Review and Meta-Analysis of Randomized Controlled Trials and Prospective Cohort Studies

**DOI:** 10.3389/fphar.2019.00773

**Published:** 2019-07-10

**Authors:** Yunsong Wang, Haichen Lv, Daobo Li, Cheng Chen, Guangming Gu, Yang Sun, Xiaolei Yang, Ying Liu, Fengqi Fang, Jiwei Liu, Gary Tse, Yunlong Xia

**Affiliations:** ^1^Department of Cardiology, The First Affiliated Hospital of Dalian Medical University, Dalian, China; ^2^Department of Oncology, The First Affiliated Hospital of Dalian Medical University, Dalian, China; ^3^Li Ka Shing Institute of Health Sciences, Faculty of Medicine, Chinese University of Hong Kong, Hong Kong, Hong Kong; ^4^Department of Medicine and Therapeutics, Faculty of Medicine, Chinese University of Hong Kong, Hong Kong, Hong Kong

**Keywords:** direct oral anticoagulants (DOACs), cancer-associated thrombosis (CAT), secondary prevention, meta-analysis, randomized controlled trials (RCTs), prospective cohort studies

## Abstract

**Background:** Venous thromboembolism (VTE) is a common complication in patients with cancer. Direct oral anticoagulants (DOACs) have been proved to be effective on anticoagulation therapy in many diseases. However, the efficacy and the safety of DOACs in the secondary prevention of cancer-associated thrombosis (CAT) remain unclear. To assess the value of DOACs in patients with CAT, we performed a systematic review and meta-analysis of randomized controlled trials and prospective cohort studies.

**Methods:** Medline, Embase, and the Cochrane Library were searched from their earliest date through to June 2018. Two investigators independently assessed eligibility. Data were extracted by one investigator and verified by the second investigator. The efficacy outcome of this study was recurrent VTE, whereas the safety outcome was major and clinically relevant nonmajor bleeding. Relative risks (RRs) and their corresponding 95% confidence interval (CI) were determined. To pool the results, the Mantel–Haenszel fixed-effects or random-effects models were used.

**Results:** A total of nine articles (six randomized controlled trials and three prospective studies) involving 2,697 patients with CAT who were prescribed DOACs (apixaban, edoxaban, rivaroxaban, or dabigatran) and 2,852 patients who were prescribed traditional anticoagulants [vitamin K antagonists (VKAs), low molecular weight heparin (LMWH), dalteparin, or enoxaparin] were compared. VTE recurrence in the DOAC group was significantly lower than that observed in the traditional anticoagulant group (RR: 0.60; 95%CI: 0.49–0.75; *I* ^2^: 0%; *p* < 0.00001). No significant difference in bleeding risk between both groups was found (RR: 0.95; 95%CI: 0.67–1.36; *I* ^2^: 75%; *p* = 0.79).

**Conclusions:** Our findings showed that anticoagulant therapy with DOACs may be more effective than traditional anticoagulants to prevent recurrent VTE in patients with CAT, while the safety of DOACs may be equal to that of traditional anticoagulants. These findings support the use of DOACs as the first-line therapy for secondary prevention of CAT in most cancer patients.

## Introduction

Venous thromboembolism (VTE), which includes deep vein thrombosis (DVT) and pulmonary embolism (PE), is a common complication in patients with cancer. Prior studies have suggested that the risk of VTE in cancer patients could be elevated four to seven times (Timp et al., 2013). Unfortunately, the management of cancer-associated thrombosis (CAT) is challenging, as these patients have higher risks of both recurrent VTE and major bleeding (MB) events after treatment (Prandoni et al., 2002). Nowadays, low molecular weight heparin (LMWH) is suggested to be the most useful anticoagulant for CAT (Akl et al., 2014; Farge et al., 2016), but long-term subcutaneous administration is inconvenient for many patients. Nevertheless, most clinical guidelines (2016 ACCP: American College of Chest Physicians; 2013 ASCO: American Society of Clinical Oncology; 2015 BCSH: British Committee for Standards in Haematology; 2014 ESC: European Society of Cardiology; 2011 ESMO: European Society for Medical Oncology) prefer LMWHs as the initial treatment for CAT (Lee and Peterson, 2013; Lee et al., 2015; Bach and Bauersachs, 2016; Kearon et al., 2016; Khorana et al., 2016). Vitamin K antagonists (VKAs) are also effective, but their efficacy is influenced by many external factors, requiring repeated blood taking to assess clotting.

In recent years, direct oral anticoagulants (DOACs), including factor IIa inhibitors and factor Xa inhibitors, have been proved to be effective on anticoagulation therapy in many diseases. Compared with LMWH and VKAs, these drugs have advantages such as convenience, as no therapeutic monitoring is required. Although many randomized controlled trials (RCTs) and observational studies have examined the efficacy and safety of DOACs for the secondary prevention of CAT, there are inconsistencies regarding the results of these studies, and thus, the efficacy and safety of DOACs for the secondary prevention of CAT remain unclear. Currently, DOACs are recommended as the second-line therapy for patients who are unable or unwilling to use long-term parenteral therapy (Lee and Peterson, 2013; Lee et al., 2015; Bach and Bauersachs, 2016; Kearon et al., 2016; Khorana et al., 2016). Therefore, the aim of this systematic review and meta-analysis of RCTs and prospective cohort studies is to assess the efficacy and safety of DOACs for secondary prevention of CAT.

## Materials and Methods

### Search Strategy

A holistic review of the published articles (through the end of June 2018) with limitation to humans was performed by using Medline (PubMed), Embase, and the Cochrane Library database. The search strategies and keywords were as follow: (“new oral anticoagulants” or “direct oral anticoagulants” or “DOAC” or “NOAC” or “new oral anticoagul” or “direct oral anticoagul” or “factor Xa inhibitors” or “rivaroxaban” or “apixaban” or “edoxaban” or “antithrombins” or “direct thrombin inhibitors” or “dabigatran”) and (“cancer” or “neoplasm” or “carcinoma” or “adenoma” or “adenocarcinoma” or “lymphoma” or “leukemia”) and (“venous thrombosis” or “venous thromboembolism” or “venous thrombos” or “deep venous thrombosis” or “deep venous thrombos” or “pulmonary embolism” or “pulmonary thromboembolism” or “VTE” or “DVT” or “PE”). All our search terms are applied for anywhere in the text.

### Study Eligibility

Two authors (YW and HL) independently identified studies eligible for inclusion based on an initial screen of reference titles and abstracts. Studies were considered potentially eligible for this systematic review if they met the following inclusion criteria: 1) Adult patients (age > 18 years) developed CAT and received anticoagulant therapy with DOACs (apixaban, edoxaban, rivaroxaban, or dabigatran); 2) The outcomes were recurrent VTE or major bleeding (MB) or clinically relevant nonmajor bleeding (CRNMB); 3) We only included RCTs and prospective cohort studies and excluded case reports, review articles, guidelines, editorials, meta-analyses, and retrospective studies; 4) Only articles in English were selected for the final meta-analysis; 5) Only manuscripts with extractable primary data among patients with CAT were included in the final analysis.

### Outcome Definition

The efficacy outcome of this study was defined as recurrent VTE, and the safety outcome was MB and CRNMB during patients receiving anticoagulant treatment. These recurrent VTE events could be asymptomatic or symptomatic, including deep vein thrombosis (DVT) and pulmonary embolism (PE). DVT and PE occurring in the same patient was recorded as single event. MB and CRNMB episodes were defined according to the criteria of the International Society on Thrombosis and Haemostasis (Schulman and Kearon, 2005; Kaatz et al., 2015).

### Data Extraction and Quality Assessment

Data were extracted into a standardized collection form by one investigator (YW) and verified by a second (HL). Data collected from each study included author, year of publication, study design, duration of patient follow-up, sample size, type of anticoagulation, mean age, male gender, and endpoint definition and incidence. Risk of bias for each study was using the Cochrane risk of bias tool for RCTs (Higgins et al., 2011) (**Table 1**); the evaluated domains included random sequence generation, allocation sequence concealment, blinding of participants and personnel, blinding of outcome assessment, incomplete outcome data, selective reporting, and other sources of bias. The quality of the prospective cohort studies were assessed using the Newcastle–Ottawa scale (**Table 2**); the evaluated domains included representativeness of the exposed cohort, selection of the unexposed cohort, ascertainment of exposure, outcome of interest not present at start of study, control for important factor or additional factor, assessment of outcome, follow-up long enough for outcomes to occur, and adequacy of follow-up of cohorts. The degree of bias found in the individual studies were categorized into high, moderate, or low risk of bias according to the Cochrane risk of bias tool and the Newcastle–Ottawa scale.

**Table 1:** Methodological quality of randomized controlled trials (RCTs) included in the meta-analysis*.

First author, publication year	Random sequence generation	Allocation concealment	Blinding of participants and personnel	Blinding of outcome assessment	Incomplete outcome data	Selective reporting	Other sources of bias	Quality
Raskob et al. (2018)	+	+	−	+	+	+	+	Low risk of bias
Raskob et al. (2016)	+	+	+	+	+	+	+	Low risk of bias
Agnelli et al. (2015)	?	?	+	+	+	+	+	Moderate risk of bias
Schulman et al. (2015)	−	+	+	+	+	+	+	Low risk of bias
Prins et al. (2014)	+	−	−	+	+	+	+	High risk of bias
Young et al. (2017)	+	+	−	+	+	+	+	Low risk of bias

*Cochrane Risk of Bias Tool for randomized controlled trials is used to assess bias for RCTs.

Low risk is represented by “+”,

high risk is represented by “−”,

unclear risk/insufficient information is represented by “?”.

**Table 2:** Methodological quality of cohort studies included in the meta-analysis*.

First author, publication year	Representativeness of the exposed cohort	Selection of the unexposed cohort	Ascertainment of exposure	Outcome of interest not present at start of study	Control for important factor or additional factor^†^	Assessment of outcome	Follow-up long enough for outcomes to occur^‡^	Adequacy of follow-up of cohorts^§^	Quality
McBane et al. (2016)	☆	☆	☆	☆	☆	☆	☆	☆	Low risk of bias
Angelini et al. (2018)	☆	☆	☆	☆	☆	☆	—	☆	Moderate risk of bias
Ageno et al. (2016)	☆	☆	☆	☆	☆☆	—	☆	☆	Low risk of bias

*A study could be awarded a maximum of one star for each item except for the item control for important factor or additional factor. The definition/explanation of each column of the Newcastle–Ottawa scale is available from http://www.ohri.ca/programs/clinical_epidemiology/oxford.asp.

^†^A maximum of two stars could be awarded for this item. Studies that controlled for anticoagulation treatment for at least 3 months received one star, whereas studies that controlled for other important confounders received an additional star.

^‡^A cohort study with a follow-up time >6 months was assigned one star.

^§^A cohort study with a follow-up rate >75% was assigned one star.

### Statistical Analysis

We performed meta-analysis for the efficacy and safety outcomes, assessed by relative risk (RR), and associated 95% confidence interval (CI), with a *P* < 0.05 considered statistically significant. We used the software Review Manager (RevMan, version 5.3, The Cochrane Collaboration, Copenhagen, Denmark) (DerSimonian and Laird, 1986) to create forest plots comparing RRs using the Mantel–Haenszel fixed-effects or random effects model. The Cochrane *P* value and the *I* ^2^ statistics was used to quantify the heterogeneity across the studies (Higgins et al., 2003). Statistically, heterogeneity was considered to be present when Cochrane *P* value ≤0.05, and the *I* ^2^ statistics <25%, 25–75%, and >75% to represent low, moderate, and high degree of heterogeneity, respectively. Funnel plots were used to assess for publication bias. Subgroup analyses were conducted separately for the type of factor Xa inhibitors (e.g., rivaroxaban and edoxaban) and study type (RCTs vs. prospective cohort studies). Finally, considering heterogeneity between some studies, we carried out a sensitivity analysis by excluding one study at a time sequentially to evaluate the impact of individual data set on the overall effect estimate.

## Results

### Study Selection and Characteristics

**Figure 1:**
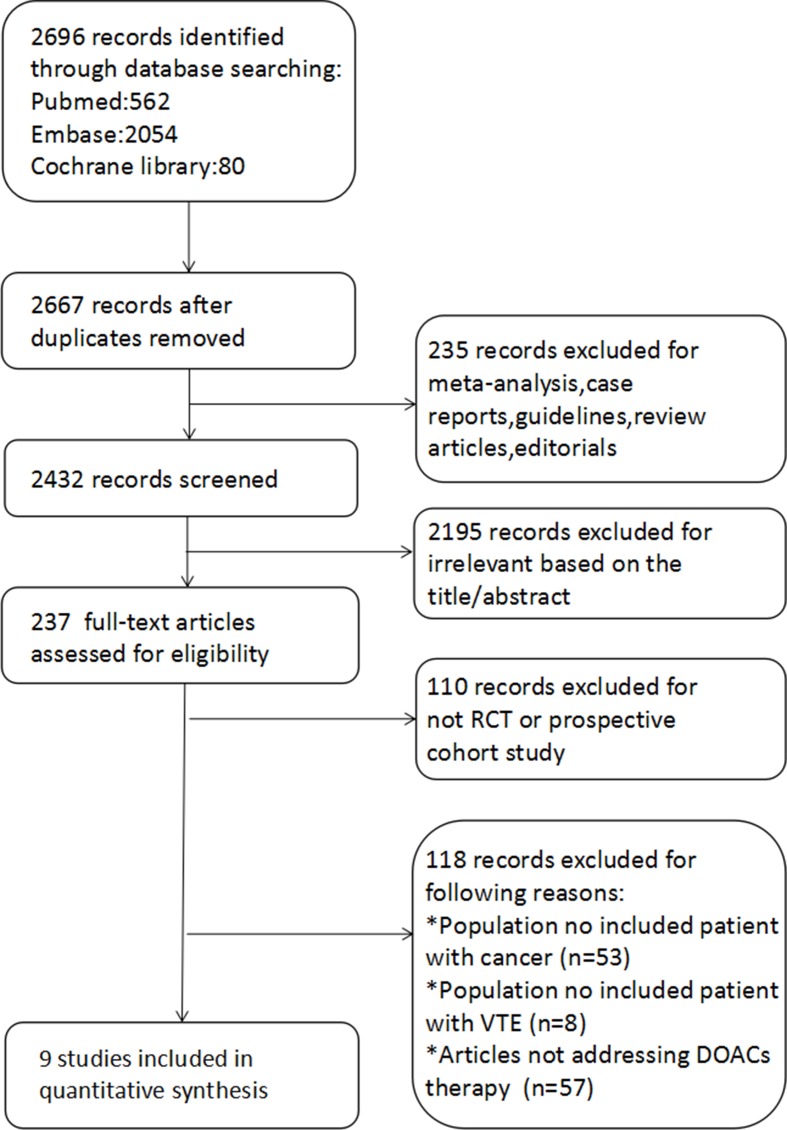
The flow diagram.

The flow diagram of the evaluation process is shown in **Figure 1**. The literature search yielded a total of 2,696 related articles. After duplicates were removed, 2,667 entries were screened further. Further 235 records were excluded, as they were case reports, review articles, guidelines, editorials, or meta-analyses. Moreover, 2,195 records were excluded, as they were not relevant to our study aim based on the title and abstract. Full-text screening led to exclusion of 110 records, as these did not take the RCT or prospective cohort study designs and 118 records as these were found to be irrelevant to our study aim.

In the end, nine articles [six RCTs (Prins et al., 2014; Agnelli et al., 2015; Schulman et al., 2015; Raskob et al., 2016; Young et al., 2017; Raskob et al., 2018) and three prospective cohort studies (Ageno et al., 2016; McBane et al., 2016; Angelini et al., 2018)] were included. A total of 2,697 patients with CAT received anticoagulant therapy with DOACs (apixaban, edoxaban, rivaroxaban, or dabigatran), and 2,852 patients received anticoagulant therapy with traditional anticoagulants (VKAs, LMWH, dalteparin, or enoxaparin). Five of the included studies recruited patients receiving anticoagulant therapy with rivaroxaban (Prins et al., 2014; Ageno et al., 2016; McBane et al., 2016; Young et al., 2017; Angelini et al., 2018), two of the studies with edoxaban (Raskob et al., 2016; Raskob et al., 2018), one with apixaban (Agnelli et al., 2015), and one with dabigatran (Schulman et al., 2015). The baseline characteristics of the studies included in this systematic review are shown in **Table 3**, while the drugs used in the assessed studies are shown in **Table 4**.

**Table 3:** Baseline characteristics.

Study	Design	Duration of study design follow-up (years)	Intervention			Outcome		
			Characteristic	DOACs	nDOACs	Endpoint	DOACs	nDOACs
Raskob et al. (2018)	RCT	12 months	Number of subjects	522	524	Recurrent VTE	41/522	59/524
			Mean age	64.3 ± 11.0 years	63.7 ± 11.7 years	MB or CRNMB	97/522	73/524
			Male gender	53.1%	50.2%			
Raskob (2016)	RCT	3–12 months	Number of subjects	650	666	Recurrent VTE	33/650	61/666
			Mean age	66y	67y	MB or CRNMB	83/572	119/569
			Male gender	48%	52%			
Agnelli et al. (2015)	RCT	6 months	Number of subjects	284	260	Recurrent VTE	5/260	16/253
			Mean age	65.5y	65.1y	MB or CRNMB	3/271	9/259
			Male gender	56.8%	60.5%			
Schulman et al. (2015)	RCT	6 months	Number of subjects	173	162	Recurrent VTE	9/173	12/162
			Mean age	63.5 ± 12.1 years	65.3 ± 12.0 years	MB or CRNMB	23/159	20/152
			Male gender	51%	55%			
Prins et al. (2014)	RCT	3–12 months	Number of subjects	584	534	Recurrent VTE	21/584	25/534
			Mean age	N/A	N/A	MB or CRNMB	73/584	71/534
			Male gender	N/A	N/A			
Young et al. (2017)	RCT	6 months	Number of subjects	203	203	Recurrent VTE	8/203	22/203
			Mean age	67y	67y	MB or CRNMB	11/203	34/203
			Male gender	53%	48%			
McBane et al. (2016)	CS	6 months	Number of subjects	135	121	Recurrent VTE	4/135	2/121
			Mean age	65 ± 14 years	66 ± 12 years	MB or CRNMB	3/135	7/121
			Male gender	50%	62%			
Angelini et al. (2018)	CS	N/A	Number of subjects	24	166	Recurrent VTE	0/24	17/166
			Mean age	N/A	N/A	MB or CRNMB	N/A	N/A
			Male gender	N/A	N/A			
Ageno et al. (2016)	CS	12 months	Number of subjects	146	223	Recurrent VTE	5/146	10/223
			Mean age	69y	68y	MB or CRNMB	2/146	8/223
			Male gender	52%	47%			

*RCT, randomized controlled trial; CS, cohort study; VTE, venous thromboembolism; MB, major bleeding; CRNMB, clinically relevant nonmajor bleeding; DOACs, direct oral anticoagulants; nDOACS, not direct oral anticoagulants.

**Table 4:** The drugs used in the assessed studies.

Study	Drugs	
	DOACs	nDOACs
Raskob et al. (2018)	Edoxaban	Dalteparin
Raskob et al. (2016)	Edoxaban	Warfarin
Agnelli et al. (2015)	Apixaban	Enoxaparin/warfarin
Schulman et al. (2015)	Dabigatran	Warfarin
Prins et al. (2014)	Rivaroxaban	Enoxaparin/warfarin
Young et al. (2017)	Rivaroxaban	LMWH
McBane et al. (2016)	Rivaroxaban	LMWH
Angelini et al. (2018)	Rivaroxaban	Enoxaparin
Ageno et al. (2016)	Rivaroxaban	Dalteparin

*RCT, randomized controlled trial; CS, cohort study; LMWH, low molecular weight heparin.

### VTE Recurrence

**Figure 2:**
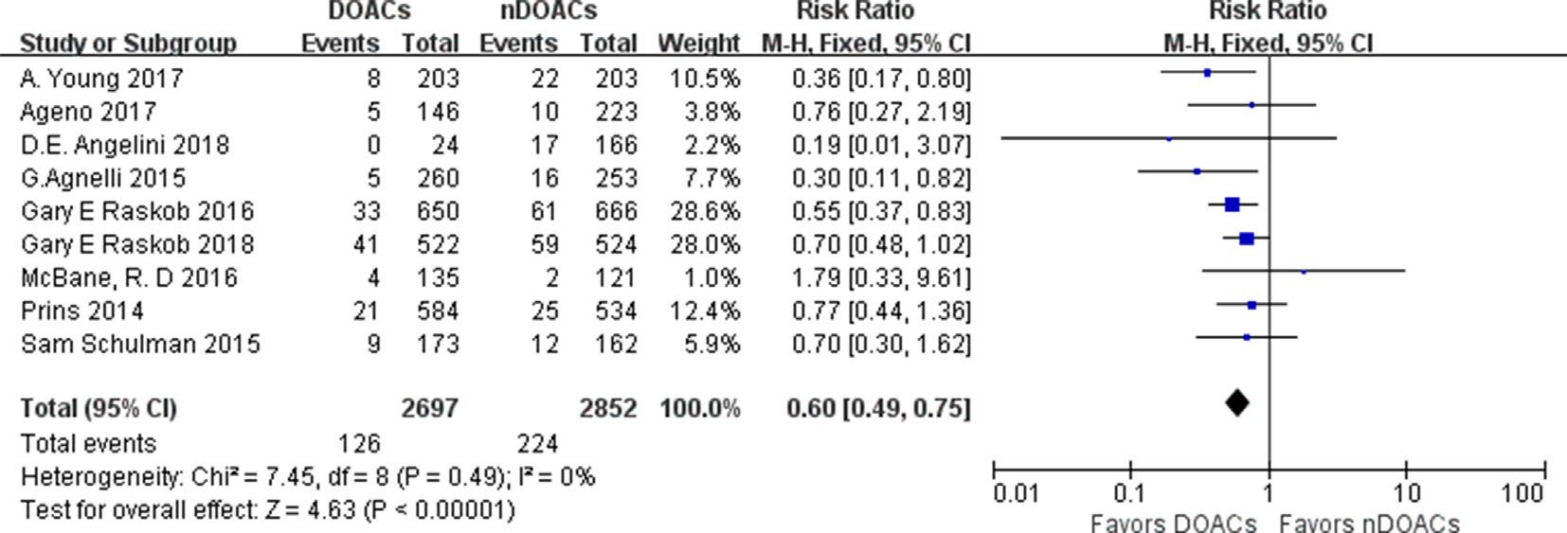
Recurrence venous thromboembolism (VTE) forest plot.

**Figure 3:**
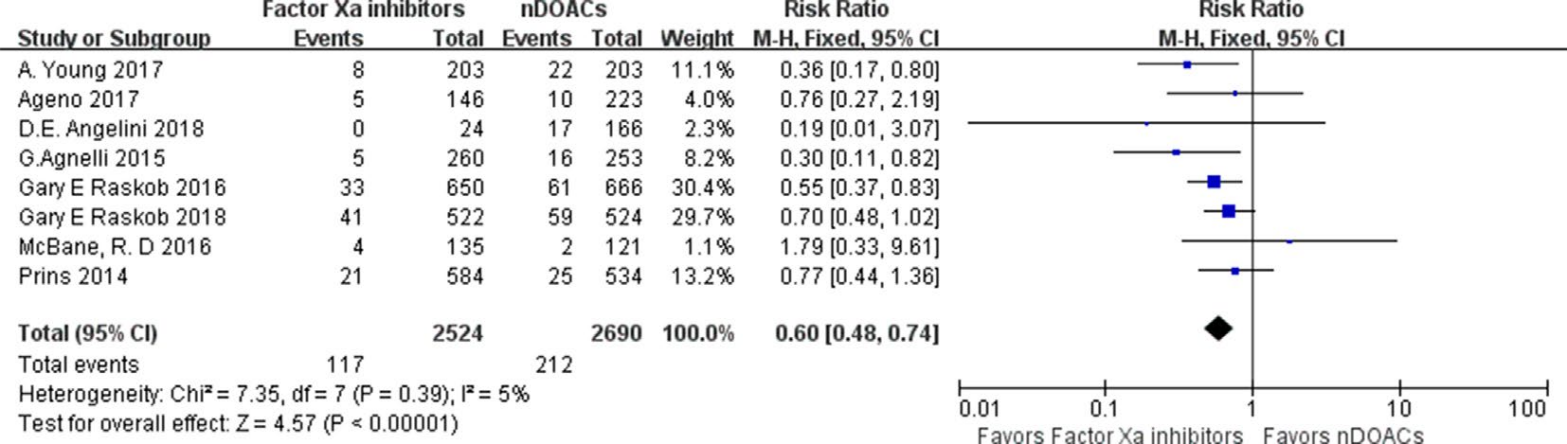
Factor Xa inhibitors recurrent VTE forest plot.

**Figure 4:**
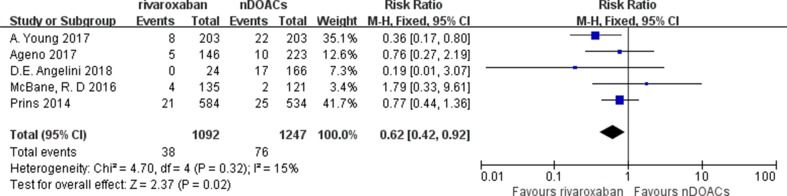
Rivaroxaban recurrent VTE forest plot.

**Figure 5:**
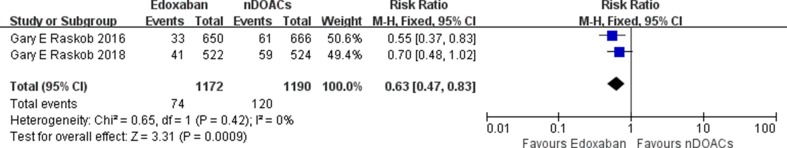
Edoxaban recurrent VTE forest plot.

VTE recurrence was evaluated in nine studies. VTE recurrence occurred in 126 of 2,697 patients (4.7%) treated with DOACs and in 224 of 2,852 patients (7.9%) treated with traditional anticoagulants. The incidence rates of VTE recurrence in survivor treated with a DOACs varied from 0 to 7.85%, and those with CAT treated with traditional anticoagulants varied from 1.6% to 11.26%. The recurrence rate of VTE in DOACs group was significantly lower than that of the traditional anticoagulant group [relative risk (RR): 0.60; 95% confidence interval (CI): 0.49–0.75; *I* ^2^: 0%; *p* < 0.00001] (**Figure 2**). Inspection of the funnel plot did not reveal publication bias (**Supplementary Figure 1**). A subgroup analysis on studies comparing factor Xa inhibitors (apixaban, edoxaban, and rivaroxaban) with traditional anticoagulants was performed, demonstrating a lower recurrence for the factor Xa Inhibitors group (RR: 0.60; 95%CI: 0.48–0.74; *I* ^2^: 5%; *p* < 0.00001) (**Figure 3**), rivaroxaban group (RR: 0.62; 95%CI: 0.42–0.92; *I* ^2^: 15%; *p* = 0.02) (**Figure 4**), and edoxaban group (RR: 0.63; 95%CI: 0.47–0.83; *I* ^2^: 0%; *p* = 0.0009) (**Figure 5**). Subgroup analysis on RCTs showed a lower recurrence for the DOACs group (**Supplementary Figure 2**) while that of the prospective cohort studies did not demonstrate a statistical significance (**Supplementary Figure 3**).

### Bleeding Events

**Figure 6:**
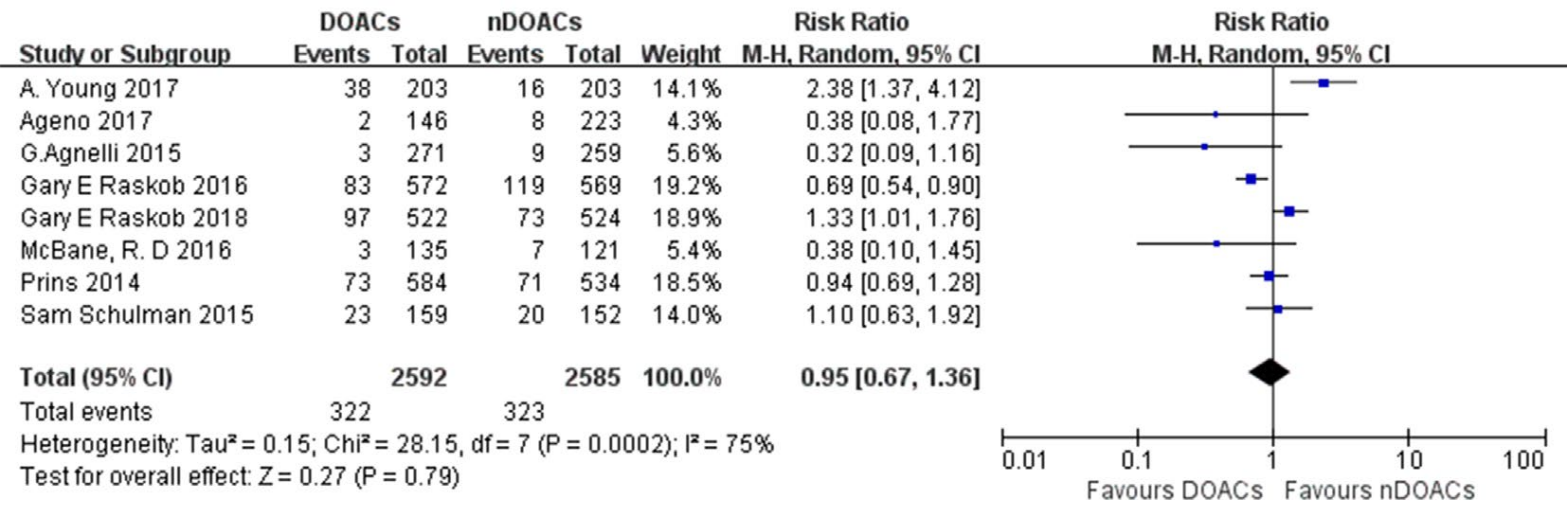
Major bleeding (MB) or clinically relevant nonmajor bleeding (CRNMB) forest plot.

**Figure 7:**
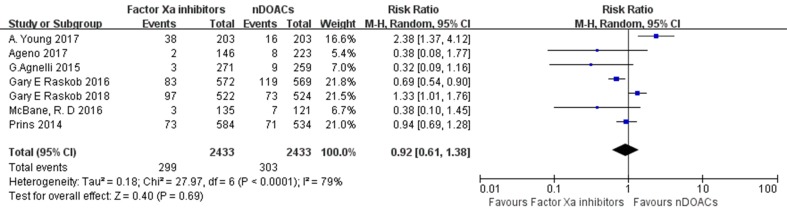
Factor Xa inhibitors MB or CRNMB forest plot.

**Figure 8:**
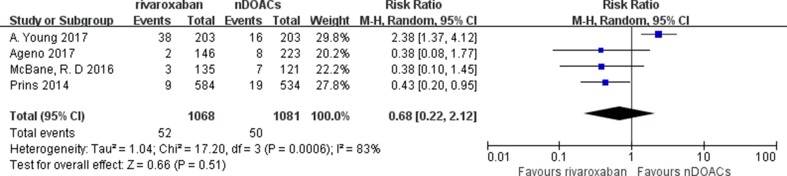
Rivaroxaban MB or CRNMB forest plot.

**Figure 9:**
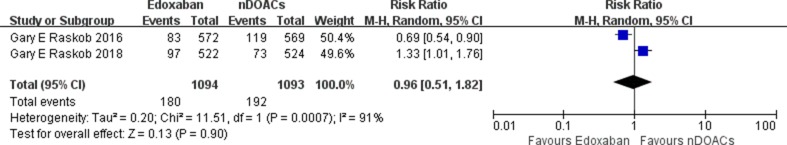
Edoxaban MB or CRNMB forest plot.

MB and CRNMB were evaluated in eight studies. Bleeding events occurred in 322 of the 2,592 patients (12.4%) treated with DOACs and in 323 of the 2,585 patients (12.5%) treated with traditional anticoagulants. The incidence rates of bleeding in patients with CAT treated with DOACs varied from 1.11 to 18.58%, and those in patients with CAT treated with traditional anticoagulants varied from 3.47 to 20.91%. No significant difference in bleeding risk was found between both groups (RR: 0.95; 95%CI: 0.67–1.36; *I* ^2^: 75%; *p* = 0.79) (**Figure 6**). There did not appear to be a publication bias across studies based on visual inspection of the funnel plots (**Supplementary Figure 4**). Three subgroup analysis on studies comparing factor Xa inhibitors (apixaban, edoxaban, and rivaroxaban), rivaroxaban, or edoxaban with not direct oral anticoagulants (nDOACs) was performed. There was no significant difference in bleeding risk between Factor Xa inhibitors group (RR: 0.92; 95%CI: 0.61–1.38; *I* ^2^: 79%; *p* = 0.69) (**Figure 7**), rivaroxaban group (RR: 0.68; 95%CI: 0.22–2.12; *I* ^2^: 83%; *p* = 0.51) **(Figure 8**), or edoxaban group (RR: 0.96; 95%CI: 0.51–1.82; *I* ^2^: 91%; *p* = 0.90) (**Figure 9**) and nDOACs group. Subgroup analysis based on study type did not reach statistical significance (RCTs: **Supplementary Figure 5**; prospective cohort studies: **Supplementary Figure 6**). Our meta-analyses demonstrated substantial heterogeneity between the studies (*I* ^2^: 75–91%).

### Sensitivity Analysis

Sensitivity analysis by the leave-one-out method was conducted to evaluate the impact of individual data set on the overall outcome. No individual study, when excluded, resulted in significant alterations in any of the study outcomes.

## Discussion

The main findings of this systematic review and meta-analysis are that anticoagulant therapy with DOACs is more effective than traditional anticoagulants to prevent recurrent VTE in patients with CAT, while the safety of DOACs is equal to traditional anticoagulants.

Over the past years, the use of DOACs has increased significantly, offering alternative choices to traditional anticoagulants for a wide range of therapeutic indications, including non-valvular atrial fibrillation (Bhardwaj et al., 2018) and VTE (Tse et al., 2018). The prevalence of VTE in cancer patients is higher than that in patients who are not suffering from cancer. Cancer patients with VTE are also at a higher risk of recurrent VTE after anticoagulant therapy. However, the efficacy and safety of DOACs in cancer patients are still unclear.

A subgroup analysis of RCTs (EINSTEIN-DVT and EINSTEIN-PE) by Prins (Prins et al., 2014) published in 2014 found that rivaroxaban had a similar efficacy to prevent recurrence of VTE and reduced MB events compared with enoxaparin and VKAs in patients with CAT. A meta-analysis of RCTs, prospective and retrospective cohort studies (Li et al., 2018), found that DOACs were more effective than LMWHs for the prevention of recurrent VTE but were associated with increased risk of bleeding. A network meta-analysis (Vedovati et al., 2018), which included 12 RCTs with DOACs, VKAs, and LMWH, found that DOACs are more effective than VKAs and equal to LMWHs without significant differences in MB. However, another network meta-analysis of 13 RCTs (Sobieraj et al., 2018) found that DOACs are more effective than VKAs and LMWH in CAT but were associated with increased MB risk compared with LMWH. These different results may be due to the differences in the number, type, and quality of the studies included. Thus, there remains no consensus on the efficacy and safety of DOACs for the secondary prevention of CAT compared with traditional anticoagulants. Therefore, we conducted this study aiming to assess the value of DOACs for the secondary prevention of patients with CAT.

To our knowledge, this is the first meta-analysis summarizing the efficacy and safety of DOACs for the secondary prevention of patients with CAT including only RCTs and prospective cohort studies. RCT and prospective studies have a lower likelihood of selection bias and recall bias compared with retrospective studies. This is the reason why the latter study type was excluded. Our meta-analysis quantitatively assessed the value of DOACs in patients with CAT compared with all the traditional anticoagulants (VKAs, LMWH, dalteparin, and enoxaparin). Our results found that the recurrence rate of VTE in DOACs group was significantly lower than that in the traditional anticoagulant group and there was no significant difference in bleeding risk between the DOAC and traditional anticoagulant groups. In addition, of the nine studies we eventually included, only one assessed dabigatran (Schulman et al., 2015). Hence, we performed a subgroup analysis concerning on factor Xa inhibitors (apixaban, edoxaban, and rivaroxaban) only. Five studies evaluated the use of rivaroxaban (Prins et al., 2014; Ageno et al., 2016; McBane et al., 2016; Young et al., 2017; Angelini et al., 2018), and two studies assessed the use of edoxaban (Raskob et al., 2016; Raskob et al., 2018). There were some differences between the assessed studies, including study designs (RCTs and prospective cohort studies), drugs used, and cancer type. All of these factors could have contributed to the heterogeneity observed between the studies. Subgroup analyses were also conducted separately for RCTs and prospective cohort studies. The results of efficacy and safety were roughly the same as the main analyses, and the heterogeneity was also acceptable. Finally, sensitivity analysis by leave-one-out method did not demonstrate significant effects by any single study.

The pharmacological actions of DOACs are well established, involving the inhibition of either factor IIa or factor Xa directly. The clearance of DOACs is less affected by other factors unlike vitamin K antagonists, and DOACs are generally eliminated from the body quickly. Their onset is relatively rapid, and reversal agents are available in case of uncontrolled bleeding. Finally, the oral route of DOACs offers advantages over subcutaneous administration for patients’ convenience. In summary, these potential mechanisms make DOACs an advantage for long-term secondary prevention of VTE recurrence in patients with CAT. Thus, a number of expert consensus are now recommending DOACs for the secondary prevention of patients with CAT. Together, our systematic review and meta-analysis contributes to the literature by providing clinicians and policymakers with new insight to aid decision making for patients with CAT. Future research studies should explore the roles of DOACs for primary prevention of CAT.

## Limitations

Several limitations of this study should be noted. Firstly, only a small number of studies have been included in this systematic review and meta-analysis, with considerable differences in drugs assessed, which may have different efficacies and safety profiles. By grouping these diverse drugs into only two groups (DOACs vs traditional anticoagulants), differences between individual drugs might have been concealed, resulting in potentially skewed results. Secondly, it should be acknowledged that there were differences in baseline characteristics of the patients enrolled into each study, such as follow-up duration, sample size, age, and gender. Moreover, information on the type of cancer and previous patient medical histories were not available in all of the studies, making meta-regression analysis not possible. Thirdly, our meta-analysis demonstrated substantial heterogeneity between the studies in terms of safety (*I* ^2^: 75–91%). We have not identified a source of heterogeneity but carried out a sensitivity analysis by excluding each included study individually, sequentially to evaluate the impact of individual data set on the overall outcome. The heterogeneity in terms of safety might have reduced the robustness of our conclusion. Finally, we have not performed a meta-analysis on DOACs for primary prevention of CAT because even a fewer number of studies were published when compared with secondary prevention. This is nevertheless an important topic, which remains to be explored in future studies.

## Conclusions

Our findings showed that anticoagulant therapy with DOACs may be more effective than traditional anticoagulants to prevent recurrent VTE in patients with CAT, while the safety of DOACs may be equal to that of traditional anticoagulants. These findings support the use of DOACs as the first-line therapy for secondary prevention of CAT in most cancer patients.

## Author Contributions

This study was conceived and designed by YW and HL, and YX, YW, and HL did the data collection and data analysis and wrote the main manuscript text along with preparing the tables and figures. YX and GT supervised the data collection and data analysis and critically revised the manuscript. All authors reviewed the manuscript.

## Funding

This study was supported by grants from the National Natural Science Foundation of China (no. 81570313, no. 81700245) and the Chang Jiang Scholars Program of China (no. T2017124).

## Conflict of Interest Statement

The authors declare that the research was conducted in the absence of any commercial or financial relationships that could be construed as a potential conflict of interest.

## Supplementary Materials

Figure S1 Recurrent VTE funnel plot.

Figure S2 RCT recurrent VTE forest plot.

Figure S3 Prospective cohort studies recurrent VTE forest plot.

Figure S4 MB or CRNMB funnel plot.

Figure S5 RCT MB or CRNMB forest plot.

Figure S6 Prospective cohort studies MB or CRNMB forest plot.
