# Supplementary figures and images for: Efficacy and Safety of Direct Oral Anticoagulants for Secondary Prevention of Cancer-Associated Thrombosis: A Systematic Review and Meta-Analysis of Randomized Controlled Trials and Prospective Cohort Studies
Source: Front Pharmacol. 2019 Jul 10;10:773. doi: 10.3389/fphar.2019.00773 (PMC6635657; doi:10.3389/fphar.2019.00773)

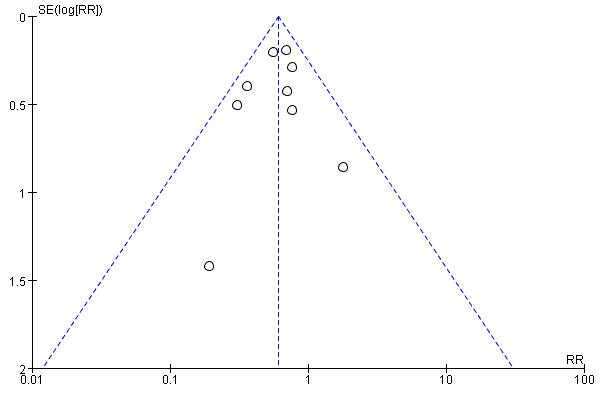

Supplement: Figure S1 — Recurrent VTE funnel plot. [file Image_1.tif]

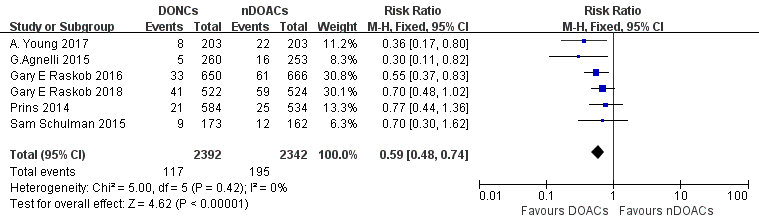

Supplement: Figure S2 — RCT recurrent VTE forest plot. [file Image_2.tif]

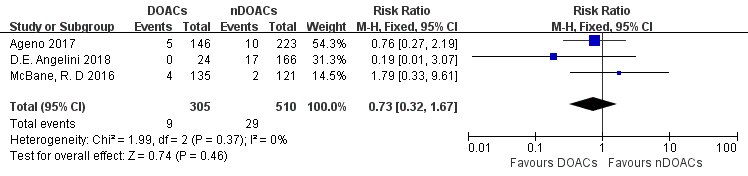

Supplement: Figure S3 — Prospective cohort studies recurrent VTE forest plot. [file Image_3.tif]

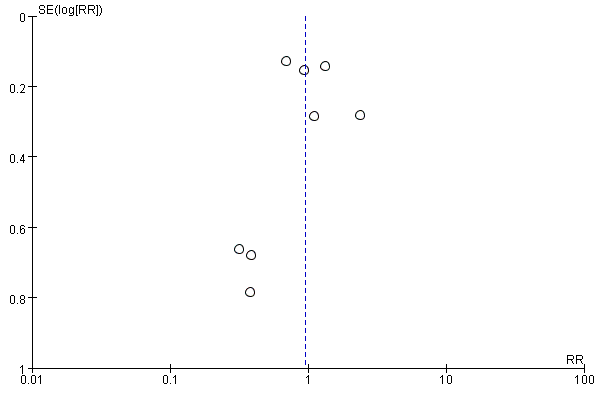

Supplement: Figure S4 — MB or CRNMB funnel plot. [file Image_4.tif]

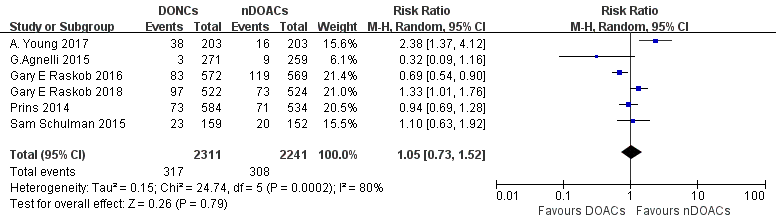

Supplement: Figure S5 — RCT MB or CRNMB forest plot. [file Image_5.tif]

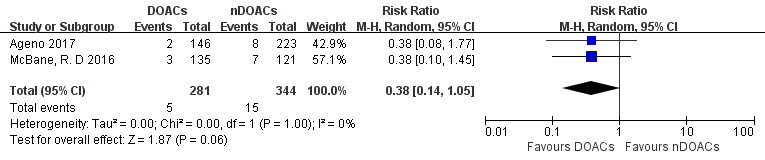

Supplement: Figure S6 — Prospective cohort studies MB or CRNMB forest plot. [file Image_6.tif]
